# Hydrogen-Rich Saline Attenuates Chronic Allodynia after Bone Fractures via Reducing Spinal CXCL1/CXCR2-Mediated Iron Accumulation in Mice

**DOI:** 10.3390/brainsci12121610

**Published:** 2022-11-24

**Authors:** Yanting Wang, Pei Wang, Cuicui Liu, Wei Chen, Pingping Wang, Lili Jiang

**Affiliations:** 1 Department of Anesthesiology, The Affiliated Hospital of Qingdao University, Qingdao University, Qingdao 266000, China; 2 Encephalopathy Clinical Center, Department of Neurology, Qingdao TCM Hospital, Qingdao 266071, China

**Keywords:** hydrogen-rich saline, bone fracture, mechanical allodynia, CXCL1, iron overload, spinal cord

## Abstract

Purpose: Neuroinflammation often initiates iron overload in the pathogenesis of neurological disorders. Chemokine-driven neuroinflammation is required for central sensitization and chronic allodynia following fractures, but specific molecular modulations are elusive. This present study explored whether hydrogen-rich saline, as one potent anti-inflammatory pharmaceutical, could alleviate fracture-caused allodynia by suppressing chemokine CXCL1 expression and iron overload. Methods: A mouse model of tibial fracture with intramedullary pinning was employed for establishing chronic allodynia. Three applications of hydrogen-rich saline (1, 5 or 10 mL/kg) were administrated intraperitoneally on a daily basis from days 4 to 6 following fractures. Spinal CXCL1 and its receptor CXCR2 levels, transferrin receptor 1 (TfR1) expression and iron concentration were examined. Recombinant CXCL1, a selective CXCR2 antagonist and an iron chelator were used for verification of mechanisms. Results: Repetitive injections of hydrogen-rich saline (5 and 10 mL/kg but not 1 mL/kg) prevent fracture-caused mechanical allodynia and cold allodynia in a dose-dependent manner. Single exposure to hydrogen-rich saline (10 mL/kg) on day 14 after orthopedic surgeries controls the established persistent fracture allodynia. Furthermore, hydrogen-rich saline therapy reduces spinal CXCL1/CXCR2 over-expression and TfR1-mediated iron accumulation in fracture mice. Spinal CXCR2 antagonism impairs allodynia and iron overload following fracture surgery. Intrathecal delivery of recombinant CXCL1 induces acute allodynia and spinal iron overload, which is reversed by hydrogen-rich saline. Moreover, iron chelation alleviates exogenous CXCL1-induced acute pain behaviors. Conclusions: These findings identify that hydrogen-rich saline confers protection against fracture-caused chronic allodynia via spinal down-modulation of CXCL1-dependent TfR1-mediated iron accumulation in mice.

## 1. Introduction

Due in large part to an increasing amount of traffic trauma and osteoporosis, the financial expenditure and epidemiological incidence of bone fractures are growing worldwide [1]. Fractures and orthopedic repairs often account for chronic pain, which features mechanical allodynia and cold allodynia and unfortunately remains to be refractory to current analgesics in clinic patients [2,3,4]. Numerous experimental findings elucidate the critical properties of the neuroinflammatory process in nociceptive synaptic plasticity in the spinal dorsal horn, which is of primary significance in persistent pro-nociception sensitization following peripheral tissue injuries, nerve trauma, cancer, chemotherapy and musculoskeletal impairments [5,6,7]. However, the detailed molecular mechanisms in the development of chronic fracture allodynia are not well investigated.

Interaction between chemokines and their receptors is considered one of the most pivotal steps in the neuroinflammatory responses underlying the pro-nociception-related process [8,9,10]. Chemokine (C-X-C motif) ligand 1 (CXCL1) which belongs to C-X-C family is identified to be implicated in the pathological pain via the tight interaction with its major receptor CXCR2 [11,12,13,14]. Specifically, the necessity of chemokine CXCL1 and its receptor CXCR2 in the recruitment of pro-inflammatory mediators has been revealed to initiate synaptic plasticity and to sustain neuropathic allodynia caused by peripheral nerve injury [11]. Moreover, CXCL1/CXCR2 cascades mediate glutamatergic neurotransmission in the pathophysiology of inflammatory hyper-nociception after acute exposure to complete Freund’s adjuvant (CFA) [12]. CXCL1 neutralizing antibody protects against bone cancer pain behaviors [13]. Additionally, pharmacological antagonism of CXCR2 impairs mechanical and thermal hyperalgesia after opioid intervention [14]. Yet, whether and how CXCL1 contributes to long-lasting fracture allodynia remains unclear.

Dysregulation of iron homeostasis is central for nociceptive synaptic transmission in remifentanil-induced hyperalgesia and fracture allodynia [15,16]. Iron overload, especially, in excitatory sensory neurons has been gradually recognized as downstream of neuroinflammation in neuropathic allodynia [17]. However, the potential link between CXCL1/CXCR2 and iron overload in pain neurocircuits is unexplored.

Recent advances have updated our understanding in the reduction of inflammation, oxidative insult and infection by molecular hydrogen in several pathological conditions [18]. Intriguingly, hydrogen-rich saline is demonstrated to attenuate opioid-caused hyper-nociception and chemotherapy-associated peripheral neuropathy in rodents [19,20]. Nevertheless, whether hydrogen-rich saline is effective against fracture allodynia through inhibition of neuroinflammation and iron overload requires further investigation.

This study investigated the possible properties of intraperitoneal (i.p.) hydrogen-rich saline in fracture-caused chronic allodynia using the mouse model of tibia bone fractures with orthopedic surgeries. The CXCL1/CXCR2 expression, iron content and iron metabolism-related protein transferrin receptor 1 (TfR1) in the spinal dorsal horn were evaluated for the verification of anti-nociceptive mechanisms of hydrogen-rich saline. Additionally, recombinant CXCL1, a selective CXCR2 antagonist and an iron chelator were employed to identify the interaction between CXCL1/CXCR2 and iron hyper-concentration in nociceptive transmission.

## 2. Materials and Methods

### 2.1. Animals

Adult C57BL/6J mice (males, 8–10 weeks old) were raised in an artificially regulated 12 h light/dark environment at 23 ± 2 °C with free access to food and water. All animals were provided from the experimental animal center of the Chinese Academy of Military Medical Science. All experimental studies and protocols were conducted in strict accordance with the National Institutes of Health Guide for the Care and Use of Laboratory Animals and approved by the Animal Ethical and Welfare Committee of The Affiliated Hospital of Qingdao University (Qingdao, China).

### 2.2. Surgical Procedure

The mouse model of tibial fracture associated postoperative allodynia was established as in previous reports [16,21]. In short, the animals were anesthetized with sevoflurane inhalation (induction at 3.0% and surgery at 1.5%) by a nose mask. Muscles were disassociated following an incision from the knee to the midshaft of the left tibia. After the osteotomy, a 0.38-mm stainless steel pin was inserted into the tibia intramedullary canal, and the incision was sutured with 3-0 silk. Sham operation was carried out by making the incision identically but with no tibial fracture and intramedullary pin insertion.

### 2.3. Preparation of Hydrogen-Rich Saline

Hydrogen-rich saline was produced as in a previous description [22]. Hydrogen was dissolved in normal saline (0.9% sodium chloride injection) for 6 h under high pressure (0.4 MPa) to a supersaturated level using a hydrogen-rich water producing apparatus (YUTAKA Engineering Co., Higashiosaka, Japan). The saturated hydrogen saline was stored at 4 °C under atmospheric pressure in an aluminum bag without dead volume. Additionally, hydrogen-rich saline is freshly produced daily to keep a stabilized concentration (>0.6 mmol/L).

### 2.4. Drug and Administration

A selective CXCR2 antagonist SB225002 (Tocris, Bristol, UK) and recombinant CXCL1 (Abcam, Cambridge, UK) was dissolved in 0.9% normal saline and administration was carried out via intrathecal injection. Deferoxamine (DFO, Sigma-Aldrich, St. Louis, MI, USA) was dissolved in 1% dimethyl sulfoxide (DMSO, Sigma-Aldrich, USA) for intrathecal injection. Under brief anesthesia with sevoflurane, drug injections with 30G needles through intrathecal routes were conducted at the L4-5 spinal segment [23].

### 2.5. Behavioral Tests

The baseline peripheral mechanical and cold sensitivity were tested 1 day before any experimental treatments, and all animals were habituated 2 h per day in the testing circumstance for 3 days prior to the basal pain examinations. For evaluating mechanical allodynia, the plantar surface of the left hind paw underwent stimulation with perpendicularly presented von Frey hairs, with exponentially increasing stiffness from 0.02 g to 2.56 g; Stoelting, Wood Dale, IL, USA). Then, 50% PW’s mechanical threshold was determined by the Dixon’s up and down method [16,21]. For cold allodynia assessment, two acetone applications (20 μL each) were gently applied to the left hind paw bottom using a pipette and the responses to acetone were scored: 0, no response; 1, quick withdrawal, paw stamping or flicking; 2, prolonged withdrawal or repeated flicking of the paw; 3, repeated paw flicking and licking [16,21]. For animals with bone fracture, pain behavioral tests were performed on 5 d, 7 d, 10 d, 14 d and 21 d after orthopedic surgeries. For animals with acute exposure to recombinant CXCL1, pain behavioral tests were performed at 1 h, 6 h and 12 h after intrathecal injections. An investigator blinded to the treatments collected the behavioral data.

### 2.6. ELISA Analysis

An enzyme-linked immunosorbent assay (ELISA) was used to measure the concentrations of CXCL1 (Abcam, UK), and CXCR2 (Wuhan Fine Biotech Co., Wuhan, China) in the L4-5 levels of left spinal cord [24]. Spinal cord tissues were homogenized in a lysis buffer containing protease and phosphatase inhibitors. Tissue samples were centrifuged at 12,500× *g* for 10 min and the supernatant was collected. BCA Protein Assay (Pierce) was employed to determine protein concentrations. For each reaction in a 96-well plate, 100 μg of proteins from the samples was used. All ELISA experiments followed the manufacturer’s protocol. The optical densities of samples were measured using an ELISA plate reader (Bio-Rad, Hercules, CA, USA) at a wavelength of 450 nm and the levels of CXCL21 and CXCR2 were calculated using the standard curves and normalized to the total protein levels.

### 2.7. Western Blot

All the animals were sacrificed under deep anesthesia of sevoflurane (3%). The left L3-5 segments of spinal dorsal horn were removed rapidly and homogenized in ice-cold RIPA buffer containing PMSF (Abcam, Cambridge, UK). The lysate was centrifuged, and the supernatant was collected as the total protein. The protein content was determined using the bicinchoninic acid assay method. The equivalent amount of proteins was resolved on a 10% SDS-PAGE gel, transferred to nitrocellulose membrane and probed with monoclonal mouse anti-β-actin antibody (42 KDa; 1:5000; Sigma-Aldrich) and polyclonal rabbit antibody against transferrin receptor 1 (TfR1, 1:5000; ZenBioScience, Durham, NC, USA), followed by incubation with horseradish peroxidase-conjugated secondary antibodies (1:2000, Jackson ImmunoResearch, West Grove, PA, USA). The membrane-bound secondary antibodies were visualized with enhanced chemiluminescence (Thermo Scientific, Rockford, IL, USA) and quantified using Gene Tools Match software (Syngene, Cambridge, UK).

### 2.8. Iron Content Assay

The iron content of the spinal dorsal horn was detected by flame atomic absorption spectrophotometer (Shimadzu AA-6800, Kyoto, Japan) at 248.3 nm with the digestion of tissues [19]. To obtain dry mass, samples (0.1–0.2 g) were dried at 60 °C for 12 h, digested with 1 mL nitric acid (60%) at 100 °C in a water bath for 2 h, which was further continued for another 0.5 h in boiling after addition of hydrogen peroxide (0.5 mL). The totally dissolved residues were diluted to 10 mL with double distilled water before calculation. Atomic iron levels were analyzed by comparing the absorbance to a wide range of standard concentrations of FeSO_4_.

### 2.9. Statistical Analysis

All data were analyzed with SPSS 19.0 (SPSS, Chicago, IL, USA). Results are shown in box-and-whiskers plots with the “box” depicting the median and the 25th and 75th quartiles and the “whiskers” showing the 5th and 95th percentiles. Individual data points were superimposed on the box-and-whiskers plots. Behavioral data analysis was carried out by two-way ANOVA with post hoc Bonferroni test. Differences of biochemical data were compared using one-way ANOVA with post hoc Bonferroni test. The criterion for statistical significance was *p* < 0.05.

## 3. Results

### 3.1. Hydrogen-Rich Saline Reduces the Generation and Maintenance of Mechanical Allodynia and Cold Allodynia Following Tibial Fracture and Orthopedic Surgeries

**Figure 1:**
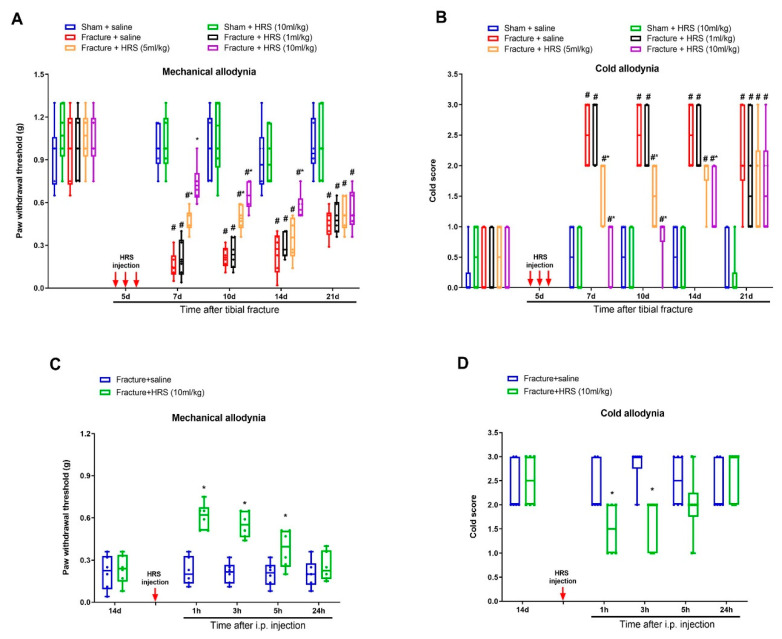
Systemic administration of hydrogen-rich saline reduces fracture-caused chronic allodynia. Intraperitoneal (i.p.) hydrogen-rich saline (HRS, 1, 5 and 10 mL/kg) was injected daily for 3 consecutive days on days 4, 5 and 6 (indicated by red arrows) after tibial fractures. The development of mechanical allodynia was assessed by paw withdrawal mechanical threshold (**A**) in von Frey test after fracture and HRS injections. The development of cold allodynia was assessed by cold response scoring (**B**) in acetone test after fracture and HRS injections. (**C**,**D**) A single HRS injection (i.p., 10 mL/kg) on day 14 after orthopedic surgeries reduces the established mechanical allodynia and cold allodynia. Results are expressed as medians with interquartile ranges and individual data plots (*n* = 6). All behavioral data are analyzed by two-way ANOVA with Bonferroni post hoc comparisons. # *p* < 0.05 vs. group Sham + saline, * *p* < 0.05 vs. group Fracture + saline.

First, there were no significant differences in basal mechanical sensitivity and cold response to acetone between sham and fracture animals (*p* > 0.05, *n* = 6, Figure 1A,B). Sham surgeries failed to induce any remarkable alternation in postoperative paw withdrawal threshold and cold response scores as compared to baseline (*p* > 0.05, *n* = 6, Figure 1A,B). Strikingly, tibial fractures generated long-lasting (>21 d, the last examination) post-surgical allodynia (mechanical allodynia and cold allodynia), as represented by marked decrease in paw withdrawal mechanical threshold (Figure 1A) and elevation in cold response to acetone (Figure 1B) after orthopedic repairs (tibial intramedullary pin insertion).

After the successful establishment of persistent allodynia by tibial fracture, we investigated the potential effect of hydrogen-rich saline on chronic fracture allodynia. First, mice received three intraperitoneal injections of hydrogen-rich saline (1, 5 and 10 mL/kg) daily on days 4, 5 and 6 (in the early phase) following orthopedic operations. Von Frey and the acetone tests revealed that hydrogen-rich saline at the dose of 5 and 10 mL/kg (but not 1 mL/kg) prevented the production of fracture-caused mechanical allodynia and cold allodynia, as demonstrated by the long-lasting increase in paw withdrawal mechanical threshold (F (5, 150) = 70.53, *p* < 0.0001, *n* = 6, two-way ANOVA, Figure 1A) and the decrease in cold scores (F (5, 150) = 48.25, *p* < 0.0001, *n* = 6, two-way ANOVA, Figure 1B) in a dose-dependent manner in fracture mice. The robust anti-allodynia was sustained for 1 week after termination of the third treatment. Furthermore, a single injection of hydrogen-rich saline (i.p., 10 mL/kg) on 14 days (in the late phase) after fracture procedures exhibited a transient and remarkable restraint of the established mechanical allodynia for 5 h (F (1, 50) = 55.99, *p* < 0.0001, *n* = 6, two-way ANOVA, Figure 1C) and cold allodynia for 1 h (F (1, 50) = 8.571, *p* = 0.0051, *n* = 6, two-way ANOVA, Figure 1D). Taken together, the behavioral data manifest that hydrogen-rich saline protects against the induction and persistence of fracture-caused chronic allodynia in mice.

### 3.2. Hydrogen-Rich Saline Reduces the Spinal CXCL1/CXCR2 Expressions and Tfr1-Dependent Iron Accumulation upon Tibial Fracture Procedures in Mice

**Figure 2:**
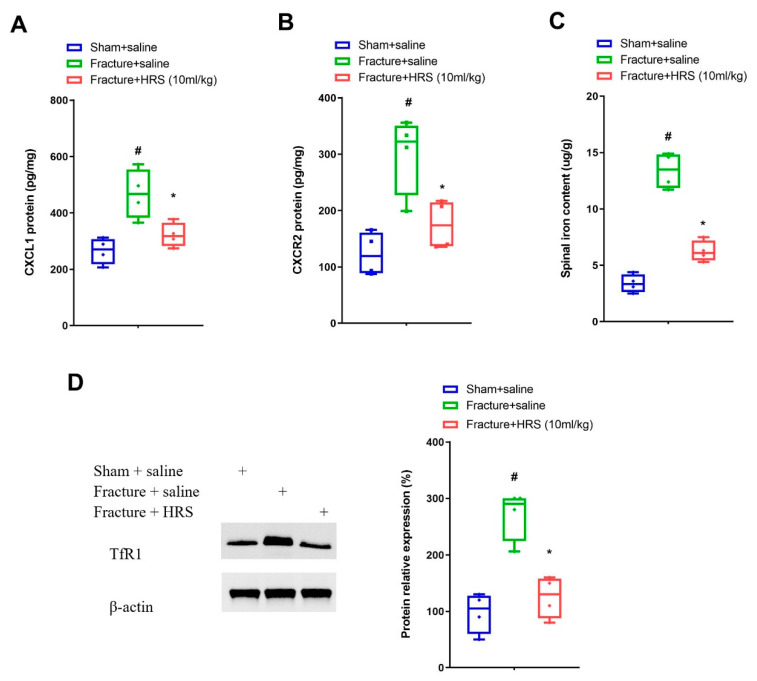
Repetitive injections of HRS reduce spinal CXCL1/CXCR2 expression and TfR1-dependent iron overload. Intraperitoneal (i.p.) hydrogen-rich saline (HRS, 10 mL/kg) was injected daily for 3 consecutive days on days 4, 5 and 6 (indicated by red arrows) after tibial fractures. (**A**,**B**) Elisa assay showed the changes of spinal CXCL1 and CXCR2 levels on day 7 following tibial fracture and HRS injections, respectively. (**C**) Spinal iron concentration on day 7 following tibial fracture and HRS injections was measured using atomic absorption spectrophotometer. (**D**) Western blot showed the changes of spinal TfR1 on day 7 following tibial fracture and HRS injections. Results are expressed as medians with interquartile ranges and individual data plots (*n* = 4). The biochemical data are analyzed by one-way ANOVA with Bonferroni post hoc comparisons. # *p* < 0.05 vs. group Sham + saline, * *p* < 0.05 vs. group Fracture + saline.

In general, expression variations in chemokines and their receptors in the spinal dorsal horn are a key step for the pathophysiology of fracture-caused chronic allodynia developments [24,25,26]. Elisa analyses represented a considerable elevation in the protein levels of CXCL1 and its receptor CXCR2 on day 7 following fracture procedures in mice (*p* < 0.05, *n* = 4, one-way ANOVA; Figure 2A,B). Moreover, these upregulations of CXCL1 and CXCR2 expression were abrogated by hydrogen-rich saline (i.p., 10 mL/kg) pre-treatment (*p* < 0.05, *n* = 4, one-way ANOVA; Figure 2A,B). Simultaneously, spinal TfR1 protein amounts and iron contents were enhanced on day 7 following fracture surgeries, whereas these alternations were reversed by hydrogen-rich saline therapy (*p* < 0.05, *n* = 4, one-way ANOVA; Figure 2C,D). These biochemical data suggested that analgesic properties of hydrogen-rich saline in fracture animals might be through down-modulating spinal neuroinflammation and iron overload.

### 3.3. CXCR2 Antagonism Reduces Chronic Allodynia Behaviors and Spinal Tfr1-Dependent Iron Overload after Fracture Procedures

**Figure 3:**
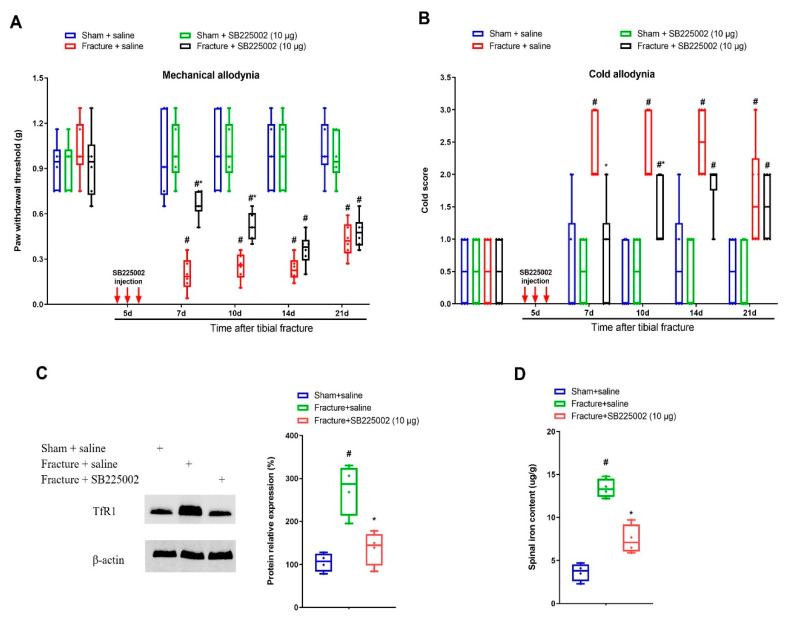
Spinal CXCR2 antagonism prevents fracture-caused chronic allodynia. The selective CXCR2 antagonist SB225002 (10 μg) was intrathecally (i.t.) injected daily for 3 consecutive days on days 4, 5 and 6 (indicated by red arrows) after tibial fractures. The development of mechanical allodynia was assessed by paw withdrawal mechanical threshold (**A**) in von Frey test after fracture and SB225002 injections. The development of cold allodynia was assessed by cold response scoring (**B**) in acetone test after fracture and SB225002 injections. All behavioral data (*n* = 6) are analyzed by two-way ANOVA with Bonferroni post hoc comparisons. (**C**) Spinal iron concentration on day 7 following tibial fracture and SB225002 injections was measured using atomic absorption spectrophotometer. (**D**) Western blot showed the changes of spinal TfR1 on day 7 following tibial fracture and SB225002 injections. The biochemical data (*n* = 4) are analyzed by one-way ANOVA with Bonferroni post hoc comparisons. Results are expressed as medians with interquartile ranges and individual data plots. # *p* < 0.05 vs. group Sham + saline, * *p* < 0.05 vs. group Fracture + saline.

**Figure 4:**
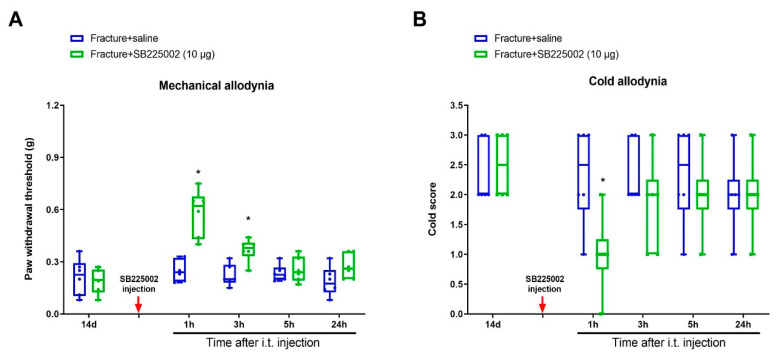
Spinal CXCR2 antagonism reduces the established fracture-caused chronic allodynia. The selective CXCR2 antagonist SB225002 (10 μg) was intrathecally (i.p.) injected on day 14 after orthopedic surgeries. (**A**,**B**) Single injection of SB225002 reduces the established mechanical allodynia and cold allodynia. Results are expressed as medians with interquartile ranges and individual data plots (*n* = 6). All behavioral data are analyzed by two-way ANOVA with Bonferroni post hoc comparisons. * *p* < 0.05 vs. group Fracture + saline.

To investigate whether CXCL1/CXCR2 cascade is important in chronic allodynia phenotypes following fracture operations, the selective CXCR2 antagonist SB225002 was employed. First, mice received three intrathecal injections of SB225002 (10 μg) daily on days 4, 5 and 6 following tibial fracture and intramedullary pin insertion. No changes in basal mechanical and cold peripheral sensitivities were witnessed in sham mice with SB225002 administration (Figure 3A,B). Intriguingly, the von Frey test detected that repeated delivery of SB225002 compromised the reduction of paw withdrawal mechanical threshold due to fracture procedures (F (3, 100) = 82.64, *p* < 0.0001, *n* = 6, two-way ANOVA; Figure 3A). Similarly, the acetone test showed that fracture-associated cold allodynia was prevented by SB225002 pre-treatment (F (3, 100) = 36.51, *p* < 0.0001, *n* = 6, two-way ANOVA; Figure 3B). The evident anti-allodynia was seen from day 1 following three injections and continued for over 1 week. More importantly, SB225002 therapy restrained the spinal over-expression of TfR1 and iron accumulation (*p* < 0.05, *n* = 4, one-way ANOVA; Figure 3C,D) on day 7 following orthopedic procedures. Additionally, on day 14 after fracture, single application of SB225002 (10 μg) attenuated the established mechanical allodynia for 3 h (F (1, 50) = 28.6, *p* < 0.0001, *n* = 6, two-way ANOVA; Figure 4A) and cold allodynia for 1 h (F (1, 50) = 5.538, *p* = 0.0226, *n* = 6, two-way ANOVA; Figure 4B). As a result, these specified data suggest that CXCL1/CXCR2 contributes to fracture-caused chronic allodynia via spinal regulation of TfR1-dependent iron accumulation.

### 3.4. Hydrogen-Rich Saline Impairs Exogenous CXCL1-Elicited Acute Allodynia Behaviors and Spinal Iron Overload

**Figure 5:**
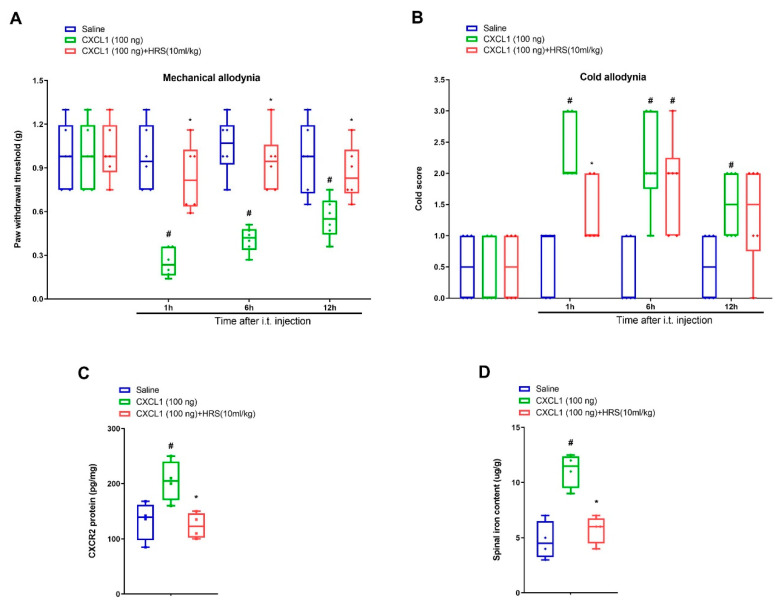
Exogenous CXCL1-evoked acute allodynia behaviors are ameliorated by systemic hydrogen-rich saline therapy. Recombinant CXCL1 (100 ng) was intrathecally injected in naïve animals. Intraperitoneal (i.p.) hydrogen-rich saline (HRS, 10 mL/kg) was injected 1 h prior to CXCL1 exposure. The paw withdrawal threshold (**A**) and cold responses to acetone (**B**) were documented following HRS and CXCL1 co-administration. The behavioral data (*n* = 6) are analyzed by two-way ANOVA with Bonferroni post hoc comparisons. (**C**) Elisa assay showed the changes of spinal CXCR2 levels on 6 h following HRS and CXCL1 co-administration. (**D**) Spinal iron concentration on 6 h following HRS and CXCL1 co-administration was measured using atomic absorption spectrophotometer. The biochemical data (*n* = 4) are analyzed by one-way ANOVA with Bonferroni post hoc comparisons. Results are expressed as medians with interquartile ranges and individual data plots. # *p* < 0.05 vs. group Saline, * *p* < 0.05 vs. group CXCL1 (100 ng).

Further, recombinant CXCL1 was utilized for determining whether CXCL1/CXCR2 cascade was implicated in nociception sensation and hydrogen-rich saline analgesia. Notably, intrathecal delivery of recombinant CXCL1 (100 ng) evoked a robust decrease in paw withdrawal mechanical threshold and increase in cold response to acetone in naive mice from 1 h to 12 h after spinal application (*p* < 0.05, *n* = 6, two-way ANOVA; Figure 5A,B). Strikingly, systemic hydrogen-rich saline (i.p., 10 mL/kg) therapy overtly ameliorated these transient pain phenotypes including mechanical allodynia (F (2, 60) = 36.54, *p* < 0.0001, *n* = 6, two-way ANOVA; Figure 5A) and cold allodynia (F (2, 60) = 20.46, *p* < 0.0001, *n* = 6, two-way ANOVA; Figure 5B). Additionally, hydrogen-rich saline reduced exogenous CXCL1-induced the spinal CXCR2 over-expression (F (2, 9) = 7.689, *p* = 0.0113, *n* = 4, one-way ANOVA; Figure 5C) and iron overload (F (2, 9) = 20.46, *p* = 0.0004, *n* = 4, one-way ANOVA; Figure 5D). Collectively, these specified findings identify the involvement of CXCL1/CXCR2 in hydrogen-rich saline anti-nociception in the mouse model of tibial fracture.

### 3.5. Exogenous CXCL1-Elicited Acute Allodynia Behaviors Are Reversed by Iron Chelation

**Figure 6:**
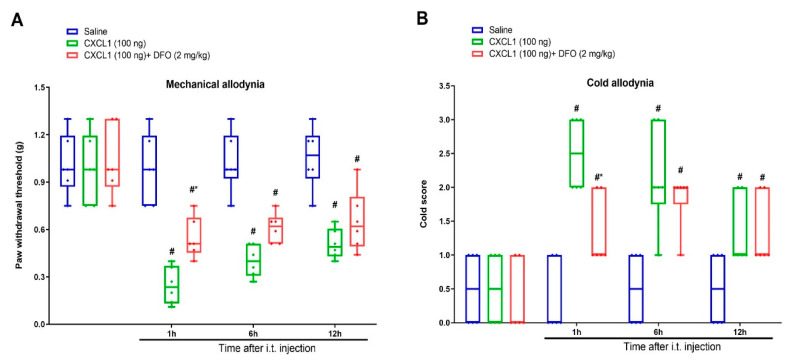
Spinal iron chelation prevents exogenous CXCL1-evoked acute allodynia behaviors. Recombinant CXCL1 (100 ng) was intrathecally injected in naïve animals. Intrathecal iron chelator DFO (2 mg/kg) was injected 1 h prior to CXCL1 exposure. The paw withdrawal threshold (**A**) and cold responses to acetone (**B**) were documented following DFO and CXCL1 co-administration. The behavioral data (*n* = 6) are analyzed by two-way ANOVA with Bonferroni post hoc comparisons. Results are expressed as medians with interquartile ranges and individual data plots. # *p* < 0.05 vs. group Saline, * *p* < 0.05 vs. group CXCL1 (100 ng).

Finally, the iron chelator DFO was employed to characterize whether iron overload is the important downstream step of CXCL1/CXCR2 cascade in central pain sensitization. DFO (2 mg/kg) was intrathecally injected 1 h prior to recombinant CXCL1 (100 ng) administration. Interestingly, we found that DFO reduced exogenous CXCL1-evoked mechanical allodynia and cold allodynia, as indicated by a dramatic elevation of paw withdrawal mechanical threshold (F (2, 60) = 49.4, *p* < 0.0001, *n* = 6, two-way ANOVA; Figure 6A) and reduction of cold response to acetone in animals with spinal CXCL1 application (F (2, 60) = 28.22, *p* < 0.0001, *n* = 6, two-way ANOVA; Figure 6B). Collectively, these specified findings recapitulate the unrecognized and critical link between CXCL1/CXCR2 cascade and iron overload in spinal nociception transmission.

## 4. Discussion

The present study, for the first time, reports that hydrogen-rich saline alleviates tibial fracture-caused mechanical allodynia and cold allodynia through spinal reduction of CXCL1/CXCR2 expression and iron overload. Furthermore, it is indicated that pharmacological inhibition of CXCL1/CXCR2 attenuates chronic fracture allodynia through down-regulating TfR1-dependent iron overload in the spinal dorsal horn. Intrathecal (spinal) exposure to exogenous CXCL1 elicits acute allodynia behaviors and spinal CXCR2 over-expression and iron overload, which was impaired by systemic hydrogen-rich saline therapy. Moreover, spinal iron chelation prevents CXCL1-induced acute allodynia.

The requirement of neuroinflammation in spinal dorsal horn for pro-nociceptive sensations has been well elucidated [5,6,7]. Chemokine and its receptors are key determinants during neuroinflammatory responses in acute and chronic pain with different etiologies [8,9,10,24,27,28,29]. Specifically, spinal cord injury causes the increase of CXCL13 and CXCR5 expression, leading to persistent neuropathic pain [27]. Interaction of CXCL12 and CXCR4 is one of the most pivotal steps for opioid-induced behavioral hyperalgesia [28]. Complete Freund’s adjuvant elicits acute inflammatory pain and hyperactivity of NR2B-containing N-methyl D-aspartate (NMDA) receptor through CCL2/CCR2 cascades [29]. Spinal neutralization of CCL21 controls the generation and maintenance of fracture-caused postoperative allodynia via mediating neuroinflammation and neuronal excitability [24]. Herein, this is the first study reporting that tibial fracture facilitates CXCL1 and CXCR2 expression in the spinal dorsal horn after orthopedic repairs and spinal CXCR2 antagonism is effective against fracture-caused persistent allodynia, suggesting that targeting CXCL1/CXCR2 cascade may be an innovative approach for fracture allodynia relief.

Neuronal iron accumulation is indispensable for the functional plasticity of excitatory glutaminergic synapses [15,30,31]. The tight interaction between neuroinflammation and iron overload has been revealed in several pathological conditions [32]. Recently, it was also indicated that exogenous CXCL10-evoked behavioral pro-nociception and CXCR3 accumulation were impaired by iron chelation [17]. Intracellular iron homeostasis in the central nervous system is mediated by a full complement of iron proteins, TfR1 in particular [33]. Neurons uptake iron through TfR1 [33]. Our biochemical results reveal that tibial fractures cause the spinal TfR1 over-expression and iron overload in mice with chronic allodynia. Furthermore, pharmacological blockages of CXCL1/CXCR2 cascade reduce the spinal TfR1-dependent iron overload following fracture and orthopedic surgeries. Exogenous CXCL1-induced acute allodynia behaviors are also reversed by spinal therapy of iron chelation. To the best of our knowledge, the present study is the first to uncover the requirement of CXCL1/CXCR2 cascade for TfR1-dependent iron overload in pain neurocircuits. However, it is of interest to evaluate how CXCL1 signaling modulates iron overload in spinal nociception process in further experiments.

In spite of medical developments and clinical practice for many years, effective strategies for controlling chronic fracture pain remain insufficient [4,26]. Opioids have several dose-limiting side effects including hyperalgesia, tolerance, nausea, and constipation [34,35,36]. Acetaminophen and NSAIDs (non-steroidal anti-inflammatory drugs) may bring a negative influence to chronic pain patients with digestive system diseases, renal function impairment and hepatic function damage [37,38]. For this reason, alternative medicines for pain-relief are in urgent need. It is noteworthy that hydrogen exhibits potent anti-inflammatory and neuroprotective properties [18]. Molecular hydrogen inhalation alleviates hypoxic-ischemic brain injury through down-regulation of inflammation and neuronal apoptosis in rats [39]. Hydrogen gas is neuroprotective against cognitive dysfunction and inflammation in sepsis-induced encephalopathy in animals [40]. However, given that inhalation of hydrogen gas is inconvenient and dangerous in clinic use [41], hydrogen-rich saline is gradually recognized as its easy administration and safe application [18]. More importantly, systemic hydrogen-rich saline therapy has been demonstrated to be beneficial for the attenuation of pathological pain, such as nerve damage-induced neuropathic allodynia, remifentanil-induced hyperalgesia, morphine-induced antinociceptive tolerance, as well as oxaliplatin-induced neuropathic pain [19,20,22,42,43]. However, the role of hydrogen-rich saline in chronic fracture allodynia has not yet been reported.

This is the first study wherein repetitive applications of hydrogen-rich saline (5 and 10 mL/kg but not 1 mL/kg) prevent fracture-caused mechanical allodynia and cold allodynia in a dose-dependent manner. Single delivery of hydrogen-rich saline (10 mL/kg) is effective against the established fracture allodynia. Moreover, systemic hydrogen-rich saline interventions reduce the CXCL1/CXCR2 expression and TfR1-dependent iron accumulation in the spinal dorsal horn of mice with chronic fracture allodynia. Additionally, hydrogen-rich saline treatment impairs exogenous CXCL1-evoked acute allodynia behaviors and spinal iron overload. These results elucidated for the first time that systemic hydrogen-rich saline therapies protect against fracture-caused chronic allodynia via inhibiting CXCL1/CXCR2-mediated neuroinflammation and TfR1-dependent iron overload, suggesting that molecular hydrogen might be utilized for developing potential therapeutic strategies in pain conditions. However, certain concerns are raised regarding how hydrogen modulates CXCL1 cascades following fractures and orthopedic repairs. One limitation is that we did not evaluate the distribution of CXCL1, CXCR2 and TfR1 proteins in the spinal dorsal horn using immunohistochemistry staining in our fracture pain models, which should be addressed in future. In addition, previous reports revealed that divalent metal transporter 1 (DMT1) is one of the most important regulators in neural iron overload during opioid-induced acute hyperalgesia and fracture-caused chronic allodynia [15,16,19]. Given that DMT1 is also a key target of hydrogen analgesia in remifentanil-caused hyperalgesia [19], it will be interesting to study whether DMT1 is implicated in hydrogen-rich saline antinociception in our mouse model of chronic fracture allodynia.

## 5. Conclusions

In summary, the present findings highlight an innovative pharmacological property of molecular hydrogen in the amelioration of tibial fracture-caused chronic allodynia through the spinal reduction of CXCL1/CXCR2 cascade-mediated TfR1-dependent iron overload. These data also suggest that hydrogen therapy and CXCR2 antagonism may be novel and neurotherapeutic strategies for fracture patients with chronic pain.

## Data Availability

All data relevant to the research are included in the paper for figures. Data are available from the corresponding author upon reasonable request.
